# Commentary: CSF and Plasma Testosterone in Attempted Suicide

**DOI:** 10.3389/fpubh.2017.00092

**Published:** 2017-04-24

**Authors:** Leo Sher

**Affiliations:** ^1^James J. Peters Veterans’ Administration Medical Center and Icahn School of Medicine at Mount Sinai, New York, NY, USA

**Keywords:** testosterone, CSF, mood, behavior, attempted suicide

The role of testosterone in the pathophysiology of suicidal behavior remains unclear. Therefore, a recent research report “CSF and plasma testosterone in attempted suicide” by Stefansson et al. published in *Psychoneuroendocrinology* is an important and timely contribution to the field (1). The authors examined the CSF and plasma testosterone and cortisol levels in medication-free suicide attempters and healthy volunteers. The authors also assessed the relationship between the testosterone/cortisol ratio, aggressiveness, and impulsivity in suicide attempters. This research group, led by Dr. Jussi Jokinen, found that male suicide attempters had higher CSF and plasma testosterone levels than age-matched male healthy volunteers. The researchers did not observe significant differences in CSF testosterone levels between female suicide attempters and healthy female volunteers. The authors observed that in male suicide attempters, the CSF testosterone/cortisol ratio showed a significant positive correlation with impulsivity and aggressiveness. This research work is important not only because it shows that testosterone may be involved in the pathophysiology of suicidal behavior but also because it suggests a potential psychobiological mechanism: it appears that impulsivity and aggressiveness may in fact mediate the effect of testosterone on suicidality. This is consistent with my hypothesis proposed in 2012 (2). I suggested that if testosterone plays a role in the neurobiology of suicidal behavior, this connection may be related to (1) a direct effect of testosterone on suicidality; (2) a testosterone effect on mood and, consequently, suicidality; (3) a testosterone effect on cognition and, consequently, suicidality; and (4) a testosterone effect on aggression and, consequently, suicidality.

Testosterone is a steroid hormone from the androgen group (3, 4). In humans, testosterone is primarily secreted in the testicles of males and the ovaries of females. Testosterone is the principal male sex hormone. The best known neurobehavioral effects of testosterone are on sexual function and aggression (5, 6). However, there is evidence that testosterone and other androgens might be involved in the pathophysiology of mood disorders and suicidal behavior (7–15).

Most but not all investigations of the relationship between testosterone and suicidality found relations between testosterone and suicidal behavior. A systematic review and meta-analysis related to a link between testosterone and suicidality have never been conducted. At least six studies indicated that testosterone levels are associated with suicidal behavior. Roland et al. (9) examined postmortem serum testosterone levels in males who died by suicide and sudden death male victims. They found that among 23- to 45-year-old males testosterone levels were significantly higher in the suicide group compared to the sudden death group. Tripodianakis et al. (10) observed significantly lower testosterone levels in male suicide attempters compared to controls. Markianos et al. (11) compared levels of plasma testosterone in psychiatric patients who had attempted suicide by jumping, male subjects who were hospitalized after accidentally falling from a high height, and healthy controls. Compared with a healthy control group, both accident and attempt groups had lower testosterone levels. There was a trend toward lower testosterone levels in suicide attempter group compared with levels in the accident group. We found that controlling for sex testosterone levels positively correlated with the number of manic episodes and the number of suicide attempts in bipolar suicide attempters (12). We also observed that in females with bipolar disorder with at least one past suicide attempt, higher baseline testosterone levels predicted suicide attempts during the follow-up period (13). Zhang et al. (14) compared testosterone levels in a large sample of suicide attempters and a large sample of healthy controls. Male or female suicide attempters had higher testosterone levels compared to male or female controls, respectively.

However, at least two studies did not support that there is a relation between testosterone and suicide. Butterfield et al. (16) did not find a difference in testosterone levels between male veteran suicide attempters with posttraumatic stress disorder (PTSD) and male veteran non-attempters with PTSD. Perez-Rodriguez et al. (17) did not find a difference in blood testosterone levels in male suicide attempters and healthy male subjects. It looks like the balance of evidence is in favor of the view that testosterone is involved in the pathophysiology of suicidality. We should, however, remember that many negative studies are not published.

It is important to note that most of the studies of the relationship between testosterone and suicidality including the study by Stefansson et al. (1) are cross sectional, i.e., the findings cannot speak to causation. Case–control studies are retrospective by their very nature: the case patients are selected because they have developed the outcome of interest, e.g., suicide attempt (18). At the same time, case–control studies are very good for the investigation of risk factors when the outcome of interest is rare, such as suicide, as it would be difficult or impossible to recruit a prospective cohort.

To a certain extent, suicidality is a proxy of the severity of psychiatric illness. Therefore, it is somewhat difficult to attribute high testosterone levels to suicidal behavior but not to those psychiatric disorders that are closely linked to suicidality.

Even in the literature on the better established link between testosterone and aggressive behavior, there are research reports that have generated mixed findings (19–22). For example, some studies suggested that higher testosterone levels are linked to aggression in men (19–21). However, a 2014 article reported that there was a significant positive correlation between testosterone levels and violent behaviors among females, but not males (22).

It is interesting to speculate that testosterone may mediate the effect of sunshine on suicidal behavior. Seasonal variations of testosterone levels and a possible impact of sunshine on suicidality have been described in the literature (23–25).

The role of testosterone and other hormones in the neurobiology of suicide remains poorly studied and poorly understood. This is a difficult field for research, but the time is ripe for active scientific research on the psychoneuroendocrinology of suicide.

## Author Contributions

The author confirms being the sole contributor of this work and approved it for publication.

## Conflict of Interest Statement

The author declares that the research was conducted in the absence of any commercial or financial relationships that could be construed as a potential conflict of interest. The reviewer, AH, and handling editor declared their shared affiliation, and the handling editor states that the process nevertheless met the standards of a fair and objective review.
